# Patient Satisfaction with Anticoagulation for Venous Thromboembolic Disease: A Systematic Review of Oral and Parenteral Regiments

**DOI:** 10.3390/medicina62040783

**Published:** 2026-04-17

**Authors:** Eleftheria Elmina Lefkou, Anastasia Fragkaki, Maria Mirsini Miliori, Dimitra Latsou, Kalliopi Panagiotopoulou, Paraskevi Kotsi, Grigorios Gerotziafas, Maria Geitona

**Affiliations:** 1 Blood Transfusion and Haemostasis Unit, Faculty of Medicine, School of Health Sciences, University of Thessaly, 41223 Larissa, Greece; mirsinimiliori@gmail.com (M.M.M.); vkotsis@uth.gr (P.K.); 2 Department of Public Administration, School of Economics, Administration and Computer Science, Neapolis University Pafos, 8042 Pafos, Cyprus; d.latsou.1@nup.ac.cy (D.L.); teigpanag@gmail.com (K.P.); geitona@uop.gr (M.G.); 3 Psychology Faculty, Aristotle University of Thessaloniki, 54124 Thessaloniki, Greece; afragkaki@psy.auth.gr; 4 INSERM UMR_S_938, Saint-Antoine Research Center (CRSA), Sorbonne University, 75012 Paris, France; grigorios.gerotziafas@inserm.fr

**Keywords:** patient satisfaction, anticoagulation, venous thromboembolic disease, quality of life

## Abstract

*Background and Objectives*: Venous thromboembolic disease (VTE), including deep vein thrombosis (DVT) and pulmonary embolism (PE), is a major cause of morbidity and mortality worldwide and imposes a substantial financial burden on health systems due to both the direct and indirect costs of acute management and long-term complications. This systematic review aimed to assess patient satisfaction with anticoagulation therapy for VTE and to highlight potential differences according to the type of anticoagulant. The review focused on factors influencing the patient experience, such as perceived efficacy, ease of use, adverse effects, and health-related quality of life. *Materials and Methods*: A systematic review, without quantitative meta-analysis, was conducted in accordance with PRISMA 2020 guidelines. Articles were identified through searches in major databases (PubMed, Scopus, Cochrane Library and others) using keywords including “patient satisfaction”, “anticoagulation”, “venous thromboembolic disease”, and “quality of life”. In total, 21 studies published between 2009 and 2025 met the inclusion criteria. The studies assessed patient satisfaction with different types of anticoagulation, including vitamin K antagonists (VKAs), direct oral anticoagulants (DOACs), and low-molecular-weight heparin (LMWH) injections. *Results*: Across the included studies, patients generally reported higher levels of treatment satisfaction with DOACs compared with VKAs, mainly due to the absence of routine laboratory monitoring and fewer dietary restrictions. However, satisfaction varied according to age, sex, and clinical status. In specific patient populations, such as those with cancer-associated thrombosis, factors including fewer drug–drug interactions and perceptions of safety with LMWH appeared to influence treatment choice and satisfaction. Adverse effects, particularly bleeding, were identified as major drivers of dissatisfaction. Several studies suggested that higher treatment satisfaction was associated with better adherence, while quality of life appeared to improve in patients treated with DOACs in comparison with VKAs. *Conclusions*: Patient satisfaction is a critical component of successful VTE management. Overall, DOACs appear to be associated with higher treatment satisfaction than traditional therapies such as VKAs, although further high-quality research is needed to individualise anticoagulation strategies. Systematic incorporation of patient-reported satisfaction into clinical decision-making and into international guidelines may improve adherence, enhance quality of life, and ultimately increase the effectiveness of anticoagulation therapy.

## 1. Introduction

Venous thromboembolic disease (VTE), which includes deep vein thrombosis (DVT) and pulmonary embolism (PE), is the third leading cause of cardiovascular-related sudden death worldwide and an important cause of morbidity and mortality, as well as a substantial financial burden on healthcare systems due to the direct and indirect costs associated with acute and long-term management [1].

VTE management is based on anticoagulation therapy. As with any pharmacological treatment, anticoagulation offers clear clinical benefits but is also associated with risks, the most important being bleeding. Therefore, the choice of the appropriate anticoagulant, as well as the duration and dosage of therapy, requires careful individualised decision-making and close cooperation between patient and physician to ensure adherence to treatment.

The economic impact of VTE is particularly significant for health systems worldwide, driven by both the immediate costs of managing acute events and the costs of long-term complications, such as post-thrombotic syndrome (PTS) and chronic thromboembolic pulmonary hypertension (CTEPH). The average cost of hospitalisation for a patient with VTE has been estimated at approximately 8000–12,000 euros per episode, depending on the severity of the event and the occurrence of complications [2,3]. In the United States, the annual cost of VTE management has been estimated at 7–10 billion US dollars, whereas in Europe it ranges from 1.5 to 2 billion euros [4]. In patients who develop chronic complications, such as CTEPH, additional annual costs can reach 15,000–20,000 euros per patient due to long-term pharmacotherapy, possible interventions, and repeated clinical and laboratory monitoring [5].

Post-thrombotic syndrome is one of the most important chronic complications of VTE, substantially affecting patients’ quality of life and further burdening healthcare systems. The estimated annual cost per patient with PTS ranges from 2500 to 5000 euros and includes treatment of venous ulcers (which may further increase annual costs to 7000–10,000 euros per patient), the use of graduated compression stockings, repeated consultations with physicians and specialists, and diagnostic tests (e.g., ultrasound imaging) [6].

In addition to these direct costs, VTE is associated with considerable indirect costs, such as productivity loss due to disability or premature death, which may account for more than 50% of the total economic burden [7]. PTS, in particular, can lead to a marked reduction in work capacity, with indirect costs from productivity loss estimated at 10,000–15,000 euros per patient per year [7].

In clinical practice, the effectiveness of a pharmacological treatment depends not only on its pharmacodynamic properties but also on the patient’s experience with the therapy. Treatment satisfaction is commonly defined as the patient’s subjective evaluation of their experience during and after receiving a specific treatment [8]. Higher treatment satisfaction has been associated with better adherence to medical recommendations, improved clinical outcomes, fewer complaints, and better perceived quality of healthcare services [8]. Systematic assessment of patient satisfaction can also help to identify aspects of care that require improvement.

Over the past decades, the therapeutic “armamentarium” of anticoagulant drugs has expanded. In addition to traditional agents such as heparins (unfractionated and low-molecular-weight) and oral coumarin derivatives (vitamin K antagonists, VKAs, e.g., acenocoumarol), direct oral anticoagulants (DOACs) have been introduced into clinical practice. These newer agents offer potential advantages, including fixed dosing, fewer food interactions, and no routine need for laboratory monitoring, which may have a positive impact on patient satisfaction and daily life.

In the specific context of VTE, relatively few studies have assessed patient satisfaction with anticoagulation, and even fewer have directly compared different types of anticoagulant therapy in terms of their impact on patient satisfaction and health-related quality of life. Given that VTE and its complications represent a significant financial burden for health systems, early diagnosis, effective prevention, and optimised treatment can substantially reduce this burden while improving patients’ quality of life. Preventive strategies, including appropriate use of anticoagulants in high-risk patients, can reduce the incidence of DVT and its long-term complications, such as PTS.

The main objective of this systematic review is to evaluate patient satisfaction with anticoagulant therapy in the treatment of VTE and to analyse the impact of anticoagulation on patients’ quality of life. Secondary objectives are to identify key factors influencing treatment satisfaction, to compare satisfaction levels between standard anticoagulation regimens (such as VKAs and low-molecular-weight heparin, LMWH) and DOACs, and to explore the relationship between treatment satisfaction and patient-reported outcomes, including adherence and health-related quality of life.

## 2. Materials and Methods

This systematic review was conducted in accordance with the PRISMA 2020 (Preferred Reporting Items for Systematic Reviews and Meta-Analyses) guidelines [9]. The protocol was not prospectively registered in PROSPERO, which is acknowledged as a limitation in the Discussion.

### 2.1. Literature Search

A comprehensive search of the international literature was performed in the following electronic databases and search engines: PubMed, EMBASE, Cochrane Library, Google Scholar, CINAHL, and Web of Science. The search covered the period from January 2009 to December 2025. In addition, supplementary sources, including the Neapolis University Library resources, the institutional repository HFAISTOS, Europeana, and other academic repositories, were screened to identify additional relevant studies.

The search strategy combined terms related to venous thromboembolic disease (VTE), deep vein thrombosis (DVT), pulmonary embolism (PE), anticoagulation, patient satisfaction, and health-related quality of life (QoL). Search keywords included the terms “venous thromboembolic disease”, “deep vein thrombosis”, “pulmonary embolism”, “oral anticoagulation”, “vitamin K antagonists”, “direct oral anticoagulants”, “patient satisfaction”, “treatment satisfaction”, and “quality of life”. Boolean operators (AND, OR) were used to combine these terms appropriately. The full search string for PubMed is provided in the Supplementary Materials (Supplementary Table S2), and a summary of adherence to the PRISMA 2020 checklist is also presented in the Supplementary Materials (Supplementary Table S1).

### 2.2. Inclusion and Exclusion Criteria

Studies were selected according to predefined inclusion and exclusion criteria.

Inclusion criteria:

Adult population (≥18 years of age).

Studies published in the English language.

Studies evaluating patient satisfaction with anticoagulation therapy for VTE.

Randomized controlled trials, observational studies, and prospective cohort studies.

Use of validated or structured instruments to measure treatment satisfaction or closely related patient-reported outcomes.

Exclusion criteria:

Studies including pregnant women.

Case reports, single case studies, and case series.

Systematic reviews and narrative literature reviews (not included in data extraction or synthesis).

Studies focusing exclusively on atrial fibrillation.

Studies including mixed patient populations (e.g., VTE and atrial fibrillation) where data for VTE patients could not be clearly separated.

Studies of anticoagulation for other indications (e.g., mechanical heart valves).

Conference abstracts without full-text availability.

Studies without specific measures of satisfaction.

Studies without validated or clearly described satisfaction measurement tools.

Systematic reviews and simple narrative reviews were excluded from the synthesis and data extraction to avoid duplication and double-counting of data. However, their reference lists were screened to identify additional eligible primary studies.

### 2.3. Study Selection and PRISMA Flow Diagram

All retrieved records were imported into a reference management system, and duplicate entries were removed. Two reviewers independently screened titles and abstracts to identify potentially eligible studies. Full-text articles were then assessed in detail against the inclusion and exclusion criteria. Any disagreements at either screening stage were resolved through discussion, and, when necessary, by consultation with a third reviewer.

The initial search yielded 539 records. After removal of duplicates and screening of titles and abstracts, full texts were assessed for eligibility. A total of 21 studies met the final inclusion criteria and were included in the review, encompassing 17,140 patients with VTE. One study was excluded due to overlap and potential double-counting with another included study. Non-English-language studies were excluded, which may have introduced a language bias; this is addressed in the Limitations Section.

**Figure 1:**
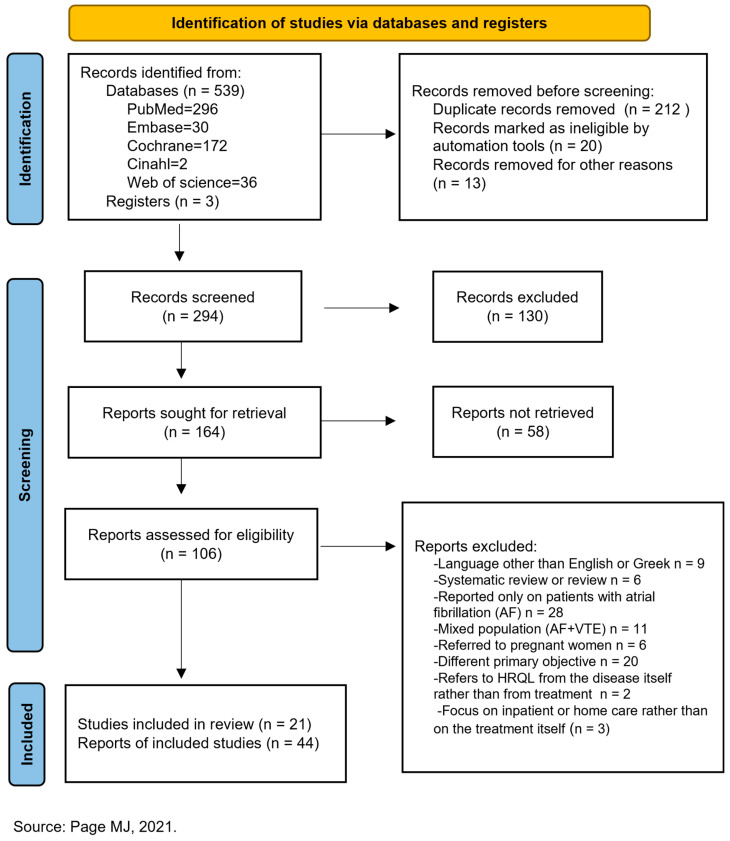
PRISMA 2020 flow diagram of the Systematic Review of the Literature on the Satisfaction of Patients with Venous Thromboembolic Disease with Anticoagulation Treatment [9].

The overall process of study identification, screening, eligibility assessment, and inclusion is illustrated in the PRISMA 2020 flow diagram (Figure 1). The PRISMA 2020 checklist is provided in Supplementary Table S1.

### 2.4. Data Extraction

Data extraction was performed using a standardized form designed for this review. For each included study, the following information was collected:

Study characteristics (design, country, setting, number of patients, duration of follow-up).

Patient demographic characteristics (e.g., age, sex).

Clinical characteristics and type of condition (e.g., DVT, PE, cancer-associated thrombosis).

Anticoagulation regimens (type of anticoagulant, doses, route of administration).

Instruments used to measure treatment satisfaction and related patient-reported outcomes (e.g., ACTS, PACT-Q, EQ-5D, SF-36).

Primary and secondary outcomes related to satisfaction, quality of life, and adherence where reported.

Duration of follow-up and key results relevant to patient satisfaction.

Data extraction was conducted independently by two reviewers, and any discrepancies were resolved by consensus.

### 2.5. Quality Assessment

The methodological quality and risk of bias of the included studies were assessed using design-appropriate tools:

- The Cochrane Risk of Bias (RoB) tool for randomised clinical trials.
- The Newcastle–Ottawa Scale (NOS; range 1–9) for cohort and other observational studies.
- The ROBINS-I tool for non-randomised interventional and observational studies.

Two reviewers independently performed the quality assessment, and disagreements were resolved by discussion or, if needed, by involving a third reviewer. A summary of the quality assessment for all 21 studies, including study title, design, and corresponding RoB, NOS score, or ROBINS-I rating, is presented in Supplementary Table S3. The overall risk of bias profile is described in the Results Section.

### 2.6. Data Synthesis

Given the substantial heterogeneity in study designs, patient populations, anticoagulation regimens, and instruments used to measure satisfaction and quality of life, a quantitative meta-analysis was not performed. Instead, a qualitative (narrative) synthesis of the findings was conducted.

Studies were grouped according to:

- Type of anticoagulant (e.g., DOACs, VKAs, LMWH).
- Study design (randomised vs. observational).
- Specific patient populations (e.g., cancer-associated VTE vs. non-cancer VTE), where applicable.
- Type of measurement instrument (e.g., ACTS, PACT-Q, generic QoL tools).

Within these groups, we summarized patterns of patient satisfaction, perceived treatment burden, quality of life, and, when reported, adherence. Where numerical satisfaction scores were presented, we reported them descriptively and clarified whether they originated from single studies or multiple studies. No statistical pooling of effect sizes, calculation of I^2^ statistics, or assessment of publication bias (e.g., funnel plots, Egger’s test) was undertaken.

### 2.7. Patient Demographics

The detailed patient demographic characteristics across the included studies are summarised in Table 1. The total number of patients in the 21 studies was 17,140, with a mean age of 59.8 years (range 46–65 years). Regarding sex distribution, most studies reported a relatively balanced proportion of men and women, with a slight overall male predominance of approximately 51%.

### 2.8. Measurement Tools for Patient Satisfaction

Table 2 provides an overview of the main instruments used across the 21 included studies to assess patient-reported outcomes related to anticoagulation therapy for VTE. The tools identified include custom surveys (focusing on specific preferences or aspects of care), treatment-specific satisfaction scales such as the Anti-Clot Treatment Scale (ACTS) and the Perception of Anticoagulant Treatment Questionnaire (PACT-Q), as well as generic quality-of-life instruments such as the EQ-5D, SF-36, and MOS SF-36.

Custom surveys were designed for individual studies to capture specific preferences or concerns (e.g., convenience, injection-related burden, monitoring requirements).

ACTS primarily evaluates the perceived benefits and burdens of anticoagulant therapy from the patient’s perspective.

PACT-Q assesses expectations, convenience, and satisfaction with anticoagulant treatment.

Generic QoL instruments (EQ-5D, SF-36, MOS SF-36) capture broader aspects of health-related quality of life, including physical, mental, and social functioning, rather than treatment satisfaction per se.

## 3. Results of the Systematic Review of Patient Satisfaction with Anticoagulation Therapy for VTE

Across the 21 included studies, most patients reported being satisfied or very satisfied with their anticoagulation therapy for VTE. In studies that reported quantitative satisfaction rates, the proportions of satisfied patients generally ranged from approximately 60% to over 90%, depending on the population, the type of anticoagulant, and the instrument used to measure satisfaction. Overall, higher satisfaction was more frequently observed among patients treated with direct oral anticoagulants (DOACs) compared with vitamin K antagonists (VKAs), whereas satisfaction with low-molecular-weight heparin (LMWH) tended to be moderate, mainly due to the burden of regular subcutaneous injections and their impact on daily life.

Several recurring themes emerged as key determinants of higher satisfaction. Patients consistently highlighted ease of use and convenience, particularly in relation to once- or twice-daily oral administration, fixed dosing schedules, and the absence of routine INR monitoring. These characteristics were perceived as reducing the overall treatment burden, especially when compared with VKAs, which require frequent dose adjustments, dietary restrictions, and regular laboratory visits. Perceived safety and limited interference with other treatments, notably in patients with cancer-associated thrombosis, further contributed to higher satisfaction, as did a favourable impact on daily functioning and health-related quality of life (QoL).

Adverse effects, especially bleeding events, were consistently identified as important drivers of dissatisfaction and treatment burden. Even when anticoagulation was clinically effective, episodes of bleeding or anxiety about bleeding risk could substantially reduce patients’ confidence in their treatment. Several studies also suggested a positive association between higher treatment satisfaction and better adherence, although the magnitude of this relationship varied and adherence was not uniformly defined or measured across studies. Taken together, the findings indicate that both perceived effectiveness and safety, alongside practical aspects of treatment, shape overall patient satisfaction with anticoagulation for VTE.

Table 3 and Table 4 summarise, respectively, the qualitative comparison of satisfaction by anticoagulant type and the key factors influencing satisfaction as reported in individual studies.

In qualitative terms, DOACs were generally associated with high satisfaction due to their simple oral administration, absence of routine INR monitoring, fewer dietary restrictions, and perceived improvements in daily functioning and QoL. VKAs were associated with moderate satisfaction, as their proven efficacy is partly offset by the inconvenience of frequent monitoring and dietary vigilance. LMWH also showed moderate satisfaction overall, remaining effective and often preferred in specific clinical contexts such as cancer-associated thrombosis, but the need for daily or twice-daily injections was perceived as burdensome and could reduce convenience and comfort.

### 3.1. Comparison Between Treatments

The included studies allowed for a qualitative comparison of patient satisfaction between different classes of anticoagulants. When DOACs were compared with VKAs, most comparative studies reported that patients preferred DOACs. This preference was largely driven by simpler dosing regimens, the absence of routine INR monitoring, and fewer dietary restrictions, which together translated into greater perceived convenience in daily life. Patients frequently described DOACs as easier to integrate into work, family, and social activities, whereas the need for regular blood tests and careful dietary management with VKAs was often experienced as restrictive.

Despite these advantages, VKAs remain appropriate and widely used in specific clinical situations, such as in patients with severe renal impairment, cost constraints, or certain guideline-defined indications. In these contexts, some patients reported acceptable levels of satisfaction when they were adequately monitored and supported, suggesting that well-organised anticoagulation services and clear communication with healthcare professionals can partially mitigate the inconvenience of VKA therapy. Satisfaction with VKAs therefore appears to depend not only on the pharmacological profile of the drug but also on the structure and quality of care.

In studies where DOACs were compared with LMWH, patients generally expressed a preference for DOACs, mainly because oral administration avoids daily injections and is perceived as less intrusive. Patients often described LMWH injections as painful, inconvenient, or difficult to self-administer over long periods, particularly in the context of chronic treatment. Nevertheless, LMWH remained a crucial option for specific patient subgroups, especially those with cancer-associated thrombosis, where efficacy data, clinician recommendations, and perceived compatibility with anticancer treatments strongly influenced both treatment choice and satisfaction. In these cases, many patients appeared willing to accept the inconvenience and discomfort of injections in exchange for what they and their physicians perceived as optimal safety and effectiveness.

Among patients with cancer-associated VTE, several studies reported that, despite the burden of injections, many patients accepted or even preferred LMWH because of perceived safety, trust in physician recommendations, and the belief that LMWH is more compatible with ongoing cancer therapies. In this population, satisfaction was often more strongly influenced by perceived effectiveness and safety than by convenience alone. These findings underline the importance of clinical context and patient priorities when comparing satisfaction between oral and parenteral anticoagulant regimens.

### 3.2. Key Factors and Patient Preferences

Across the included studies, several recurring factors were identified as central to shaping patient satisfaction and preferences. Route of administration emerged as one of the most influential determinants. In the COSIMO study, for example, route of administration accounted for 73.8% of overall treatment satisfaction, with a clear preference for oral therapy over subcutaneous injections [27]. Patients frequently described LMWH injections as painful, inconvenient, and difficult to self-administer, particularly during prolonged treatment, and these experiences negatively affected their comfort, autonomy, and daily functioning [27].

Monitoring requirements were also critical in determining satisfaction. The reduced need for frequent laboratory testing with DOACs was consistently cited as a major advantage over regimens that required regular INR checks. In the study by Keita et al., 97.8% of patients reported higher satisfaction with treatment strategies that did not require routine laboratory monitoring, highlighting how less intensive follow-up can reduce treatment burden and improve perceived convenience [23]. Conversely, the demands of frequent testing and clinic visits with VKAs were often viewed as disruptive, contributing to lower satisfaction despite good clinical efficacy.

Perceived safety and effectiveness, particularly in complex patients, further influenced treatment preferences. In cancer-associated thrombosis, for instance, Noble et al. reported that 39% of patients considered non-interference with other treatments (such as chemotherapy) and perceived safety to be major reasons for their satisfaction with LMWH [26]. In this context, many patients appeared to prioritise what they viewed as reliable protection from thrombotic events and minimal interaction with anticancer regimens, even when this meant accepting the inconvenience of injections.

Health-related QoL was another important dimension linked to satisfaction. Studies using SF-36 and other QoL instruments found that patients treated with DOACs often had better QoL scores and less treatment-related anxiety compared with those receiving VKAs [17,19]. These advantages were attributed to simpler treatment regimens, fewer dietary and lifestyle constraints, and a reduced sense of treatment burden [17,19]. At the same time, patients on LMWH, especially those with cancer, reported that injections and frequent healthcare contacts could negatively affect QoL, even when they were satisfied with the perceived safety and effectiveness of therapy.

Finally, adherence emerged as both a consequence and a determinant of satisfaction. In the study by Keita et al., higher adherence was associated with simpler treatment regimens and reduced monitoring burden, which in turn were linked to greater satisfaction [23]. Patients who found their treatment easy to integrate into daily routines and who perceived it as manageable and understandable were more likely to follow medical recommendations consistently. These findings suggest a dynamic interaction between satisfaction, QoL, and adherence, in which more convenient and patient-friendly anticoagulant strategies can support better long-term treatment engagement.

## 4. Discussion

### 4.1. Main Findings

This systematic review synthesized evidence from 21 studies including 17,140 patients with venous thromboembolic disease (VTE) who received various forms of anticoagulation. Although the studies were heterogeneous in design, populations, and outcome measures, several consistent patterns emerged that patient satisfaction with anticoagulant therapy was generally high, particularly among patients treated with direct oral anticoagulants (DOACs). Health-related quality of life (HRQoL) tended to be better in patients receiving DOACs compared with those on vitamin K antagonists (VKAs) or low-molecular-weight heparin (LMWH). Adherence appeared to be more favourable in simple regimens, required less frequent monitoring, and was better understood by patients.

These findings highlight the importance of considering not only clinical efficacy and safety, but also patient experience when selecting and managing anticoagulant therapy for VTE.

### 4.2. Patient Satisfaction with Anticoagulation

In line with the primary objective of this review, most included studies reported high or moderate levels of treatment satisfaction among patients with VTE, with DOACs generally associated with higher satisfaction than VKAs or LMWH. Rather than a single pooled estimate, satisfaction rates varied across studies depending on the population, instrument used, and comparator.

Key determinants of higher satisfaction included:

Ease of use and convenience: Once- or twice-daily oral administration, fixed dosing, and the absence of routine INR monitoring were repeatedly cited as key advantages of DOACs.
Studies such as COSIMO and others [14,27] showed that route of administration (oral vs. injectable) and simplicity of the regimen strongly influenced patient-reported satisfaction.
Reduced treatment burden: Compared with VKAs, DOACs were perceived as less intrusive in daily life, with fewer dietary restrictions and less need for frequent healthcare visits.
Cano et al., (2018) [15] reported a lower “treatment burden” with rivaroxaban compared with traditional regimens.
Specific clinical contexts: Although injections with LMWH were often experienced as inconvenient or painful, in certain subgroups—such as patients with cancer-associated thrombosis—LMWH was still frequently accepted or even preferred because of perceived safety and guideline support [14,26,27].

These findings confirm and complement the narrative results presented earlier, where DOACs emerged as the most satisfying option for many patients, while LMWH and VKAs remained important in defined clinical scenarios despite lower convenience.

### 4.3. Health-Related Quality of Life (QoL) and Its Distinction from Satisfaction

It is important to distinguish treatment satisfaction from health-related quality of life (HRQoL), even though they are related constructs. Treatment satisfaction refers primarily to the patient’s evaluation of the treatment process and its attributes (e.g., convenience, side effects, monitoring), whereas HRQoL reflects the broader impact of disease and treatment on physical, emotional, and social functioning.

In the studies included in this review:

DOACs and HRQoL: Studies using generic QoL instruments (e.g., SF-36, EQ-5D) demonstrated that patients treated with DOACs often had better or more stable HRQoL compared with those on VKAs or LMWH [17,28]. Arteaga et al. and Fang et al. (2022) [17,31] reported improvements in physical functioning and overall QoL in patients on DOACs, which were attributed to easier administration, fewer lifestyle constraints, and reduced treatment-related anxiety.

VKAs and LMWH: Patients treated with VKAs frequently reported QoL limitations related to dietary restrictions, regular INR testing, and the psychological burden of fluctuating anticoagulation levels [23,24]. LMWH, particularly in cancer-associated thrombosis (CAT), was associated with a higher emotional and practical burden due to injections, but some patients accepted this in view of perceived safety and compatibility with anticancer treatments [26,31].

Thus, higher treatment satisfaction with DOACs often coincided with better HRQoL, but the two measures are not interchangeable. Some patients may be satisfied with a treatment they perceive as effective and safe (e.g., LMWH in cancer), even if HRQoL is negatively affected by injections or frequent healthcare visits. This distinction is crucial when interpreting the literature and designing future studies.

### 4.4. Adherence and Its Relationship with Satisfaction and QoL

Adherence refers to the extent to which patients follow the prescribed treatment regimen. It is conceptually distinct from both satisfaction and QoL, but is influenced by them and, in turn, affects clinical outcomes. Across the included studies, simpler treatment regimens appeared to be associated with better adherence. Direct oral anticoagulants (DOACs), with fixed dosing and fewer monitoring requirements, were associated with better adherence in observational studies, as patients found them easier to incorporate into their daily routines [12,16,23]. In contrast, vitamin K antagonists (VKAs) require strict adherence to dosing schedules, dietary precautions, and frequent laboratory checks, which can be challenging for many patients and may lead to missed doses or treatment discontinuation [23,24].

Patient understanding and support also emerged as crucial determinants of adherence. Studies such as those by Bartoli-Abdou et al. and Marini et al. [12,32] emphasised that patient education and clear communication about the purpose, benefits, and risks of anticoagulation are critical for maintaining adherence. Patients who understood their treatment plan and felt supported by healthcare professionals generally reported a better overall experience and were more likely to adhere to their medication over time [12,32].

In special populations, such as patients with cancer-associated thrombosis, adherence to low-molecular-weight heparin (LMWH) can be negatively affected by the burden of injections, treatment-related fatigue, and concomitant oncological therapies. However, outpatient administration pathways and structured support programmes have been associated with relatively high satisfaction and acceptable adherence in these settings, suggesting that organisational factors can partially mitigate the practical challenges of long-term injectable therapy [26,31]. Overall, the evidence suggests that higher satisfaction and better HRQoL are often associated with improved adherence, but they do not guarantee it. Adherence is multifactorial and requires attention to regimen complexity, patient education, comorbidities, and psychosocial factors [12,16,23,24,26,31,32].

### 4.5. Clinical Implications and Patient-Centred Care

Traditionally, clinical practice and international guidelines for VTE treatment have focused primarily on objective indicators of efficacy and safety, such as recurrence and bleeding, with less emphasis on patient preferences, satisfaction, and HRQoL. However, health systems are increasingly moving towards a patient-centred model of care, in which patient experience and values are integral to decision-making [21,33,34,35,36,37,38,39,40,41,42,43,44,45]. The findings of this review support this evolution by showing that treatment satisfaction, QoL, and adherence are closely inter-related and can influence the real-world effectiveness of anticoagulant therapy, beyond what is captured by classical clinical endpoints.

One key implication is the integration of patient-reported outcomes (PROs) into routine care. Systematic assessment of treatment satisfaction, HRQoL, and adherence should be incorporated into everyday clinical practice and into guideline development [34,35,36,45]. Instruments such as ACTS and PACT-Q provide treatment-specific information on satisfaction and perceived burden, whereas EQ-5D and SF-36 offer broader insights into HRQoL [35,36,40,41,44]. The combined use of these tools can help clinicians identify patients who are struggling with their current regimen—whether because of practical difficulties, adverse effects, or psychological distress—and to adapt therapy or support accordingly [33,34,35,36,40,41,44,45].

Shared decision-making is another essential component of patient-centred care. Treatment decisions should take into account not only clinical characteristics and contraindications but also patient preferences regarding route of administration, monitoring requirements, lifestyle constraints, and perceived safety [37,39,43,45]. This is particularly relevant when several anticoagulation options are clinically acceptable, as in many non-cancer VTE cases where DOACs, VKAs, and, in some settings, LMWH may all be considered. Discussing the advantages and disadvantages of each option can help align the chosen regimen with patients’ values and daily life, which may in turn enhance satisfaction, adherence, and long-term outcomes [33,34,35,36,37,39,43,45].

Evolving patterns of anticoagulant use in real-world practice further underscore the relevance of patient-centred considerations. Large health insurance database studies, including 298,609 patients initiating oral anticoagulants for VTE, have shown a marked shift from warfarin to DOACs, with DOAC use increasing from 0% in 2010 to 31.9% in 2014 and 89.2% in 2020 [21,38,39]. This trend reflects not only accumulating evidence of efficacy and safety but also growing recognition of the importance of convenience, reduced monitoring, and overall patient experience in treatment selection [21,33,34,35,36,37,38,39,43,45].

Despite the advantages of DOACs, traditional agents remain necessary in specific clinical contexts. VKAs are still required for certain indications, such as severe renal impairment or cost-related constraints, and can provide effective therapy when carefully monitored [24,28]. LMWHs, with minimal drug–drug interactions and robust efficacy data, remain the standard of care in many patients with cancer-associated thrombosis, where oncological considerations and perceived safety may outweigh convenience [14,29,31]. Therefore, the goal is not to replace all traditional therapies, but to use the available evidence on satisfaction, QoL, and adherence to support individualized, preference-sensitive treatment choices within the range of clinically appropriate options [14,21,24,28,29,31,33,34,35,36,37,38,39,43,44,45].

### 4.6. Limitations

This review has several important limitations that should be considered when interpreting the findings:

Heterogeneity of studies: The included studies varied widely in design (randomised vs. observational), populations (e.g., cancer vs. non-cancer VTE), settings, follow-up durations, and instruments used to assess satisfaction, QoL, and adherence.

This heterogeneity precluded a formal quantitative meta-analysis; therefore, the synthesis is narrative and descriptive rather than statistical.

Measurement variability: Different studies used various instruments (ACTS, PACT-Q, custom surveys, EQ-5D, SF-36, MOS SF-36), which assess overlapping but not identical constructs.

As a result, direct comparison of absolute scores across studies is limited, and the conclusions rely mainly on the direction and consistency of findings.

Risk of bias and study quality: Most observational studies were judged to be at moderate risk of bias, primarily due to potential confounding and incomplete reporting.

Although the three randomized clinical trials were at low risk of bias according to the Cochrane tool, the overall evidence base is constrained by the observational nature of many studies. Detailed quality assessments are summarized in Supplementary Table S2.

Language and publication bias: Only studies published in English were included, which may introduce language bias and limit generalizability.

Conference abstracts and grey literature were excluded if full texts were unavailable, potentially favouring studies with positive or more complete reporting.

Lack of standardization of adherence measures: Adherence was not uniformly defined or measured across studies. In some cases, adherence was inferred from refill patterns, persistence, or self-report, limiting comparability.

**Protocol registration:** The review protocol was not prospectively registered (e.g., in PROSPERO), which may increase the risk of selective reporting. However, the methods were predefined and conducted in accordance with PRISMA 2020 guidelines [9].

## 5. Conclusions

In conclusion, this systematic review shows that patient satisfaction with anticoagulant therapy for VTE is generally high, particularly among patients treated with DOACs, which are consistently perceived as more convenient and less burdensome than VKAs and LMWH in many settings. Health-related quality of life also tends to be more favourable with DOACs, largely due to simplified dosing, reduced monitoring, and fewer lifestyle restrictions, although LMWH and VKAs remain indispensable for specific clinical situations and patient subgroups.

Adherence appears to be enhanced when treatments are simple, clearly explained, and aligned with patient preferences, underscoring the importance of integrating satisfaction, quality of life, and adherence into clinical decision-making and guideline development. Given that much of the underlying evidence is observational and at moderate risk of bias, the comparative advantages of different regimens for patient-reported outcomes should be interpreted with caution and viewed as supportive of patient-centred care rather than definitive. Future high-quality studies using standardised, validated instruments and including under-represented populations will be crucial to refine these conclusions and to guide more individualized anticoagulation strategies.

## Figures and Tables

**Table 1:** Patient demographic data from selected studies.

Study	Number of Patients	Sex (% Men)	Mean Age (Years)	Type of Condition	Treatment
Wong et al., 2012 [10]	227	55	50	VTE	POs vs. parenteral anticoagulant
Bamber et al., 2013 [11]	1472	52	58	DVT	Rivaroxaban vs. VKA
Bartoli-Abdou et al., 2018 [12]	525	45	65	VTE	Warfarin
Brekelmans et al., 2017 [13]	200	50	62	VTE	DOACs vs. VKA
Cajfinger et al., 2016 [14]	409	50	65	Cancer-related VTE	LMWH
Cano et al., 2018 [15]	892	48	63	VTE	Rivaroxaban vs. standard therapy
Dault et al., 2018 [16]	140	46	60	VTE	DOACs vs. VKA
Fang et al., 2022 [17]	2230	43	63	VTE	DOACs vs. warfarin
Farge et al., 2019 [18]	400	50	57	Cancer-related VTE	LMWH
Font et al., 2023 [19]	74	51	64	Cancer-related VTE	LMWH
Haac et al., 2017 [20]	232	60	48	VTE related to orthopaedic trauma	LMWH vs. aspirin
Hendriks et al., 2020 [21]	126	45	60	VTE	DOACs vs. warfarin
Hull et al., 2009 [22]	480	58	62	DVT	LMWH vs. warfarin
Keita et al., 2017 [23]	100	46	61	VTE	DOACs vs. VKA
Lutsey et al., 2023 [24]	519	17	46	VTE	DOACs vs. warfarin
Maraveyas et al., 2020 [25]	505	45	64	Cancer-related VTE	Rivaroxaban
Noble et al., 2022 [26]	100	30	57	Cancer-related VTE	LMWH vs. warfarin
Picker et al., 2021 [27]	163	49	64	Cancer-related VTE	Rivaroxaban
Prins et al., 2018 [28]	2397	51	59	PE	Rivaroxaban vs. VKA
Schulman et al., 2017 [29]	5142	53%	60	VTE	DOACs vs. VKA
Webb et al., 2019 [30]	907	48%	52	VTE	Multiple

Acronyms: VTE: Venous Thromboembolism, DVT: Deep Vein Thrombosis, DOACs: Direct Oral Anticoagulants, LMWH: Low Molecular Weight Heparin, VKA: Vitamin K Antagonists, Warfarin: A type of anticoagulant, Rivaroxaban: An anticoagulant medication.

**Table 2:** Measurement tools used to assess patient satisfaction with anticoagulation therapy.

Measurement Tool	Uses/Studies	Characteristics
Custom survey	Wong et al., 2012 [10]; Haac et al., 2017 [20]; Noble et al., 2022 [26]; Lutsey et al., 2023 [24]; Schulman et al., 2017 [29]	Study-specific questionnaires focusing on particular preferences or experiences (e.g., convenience, injection burden, monitoring).
ACTS (Anti-Clot Treatment Scale)	Bamber et al., 2013 [11]; Cano et al., 2018 [15]; Hendriks et al., 2020 [21]; Maraveyas et al., 2020 [25]; Prins et al., 2018 [28]	Evaluates the balance between perceived benefits and burdens of anticoagulant therapy (treatment burden and benefits domains).
PACT-Q (Perception of Anticoagulant Treatment Questionnaire)	Brekelmans et al., 2017 [13]; Cajfinger et al., 2016 [14]; Dault et al., 2018 [16]	Assesses patient expectations, convenience, and satisfaction with anticoagulant treatment.
EQ-5D	Bartoli-Abdou et al., 2018 [12]	Generic health-related quality of life instrument covering mobility, self-care, usual activities, pain/discomfort, and anxiety/depression.
SF-36	Fang et al., 2022 [17]; Farge et al., 2019 [18]	Measures multiple domains of health-related quality of life, including physical functioning, role limitations, bodily pain, general health, vitality, social functioning, and mental health.
MOS SF-36	Farge et al., 2019 [18]	Shorter form of the SF-36 focusing on key dimensions of health-related quality of life relevant to patients with chronic conditions.

**Table 3:** Comparison of patient satisfaction by anticoagulant type.

Therapy Type	Qualitative Level of Satisfaction	Key Comments
DOACs	Generally high	Convenience of once- or twice-daily oral dosing, absence of routine INR monitoring, fewer dietary restrictions, and perceived improvement in daily functioning and QoL.
VKAs	Moderate	Requirement for frequent INR monitoring, dose adjustments, and dietary restrictions may negatively impact convenience and perceived QoL, despite proven efficacy.
LMWH	Moderate	Effective and often preferred in specific clinical contexts (e.g., cancer-associated thrombosis), but daily or twice-daily subcutaneous injections are perceived as burdensome and may reduce convenience and comfort.

**Table 4:** Key factors influencing satisfaction (examples from individual studies).

Factor	Association with Satisfaction	Key Study
Route of administration	In the COSIMO study, 73.8% of overall treatment satisfaction was attributed to the route of administration, with a clear preference for oral therapy over injections [27].	COSIMO Study (Picker, 2021) [27]
Ease of use/monitoring	In a cross-sectional study, 97.8% of patients reported higher satisfaction with regimens that did not require frequent laboratory monitoring [23].	Keita, 2017 [23]
Safety/effectiveness in complex patients	In patients with cancer-associated thrombosis, 39% of patients indicated that non-interference with other treatments (e.g., chemotherapy) and perceived safety were major reasons for their satisfaction with LMWH [26].	Noble, 2022 [26]
Quality of life (QoL)	Studies using SF-36 and other QoL instruments reported that patients on DOACs had better QoL scores and less treatment-related anxiety compared with those on VKAs, attributed to simpler regimens and fewer lifestyle constraints [17,19].	Fang, 2022 [17]; Font, 2023 [19]

## Data Availability

The data presented in this study are available within the article.

## Supplementary Materials

The following supporting information can be downloaded at: https://www.mdpi.com/article/10.3390/medicina62040783/s1, Table S1: PRISMA 2020 Checklist; Table S2: The full search string for PubMed; Table S3: Quality assessment results.
