# Supplementary material for: Patient Satisfaction with Anticoagulation for Venous Thromboembolic Disease: A Systematic Review of Oral and Parenteral Regiments
Source: Medicina (Kaunas). 2026 Apr 17;62(4):783. doi: 10.3390/medicina62040783 (PMC13117933; doi:10.3390/medicina62040783)
Supplement: Supplementary file 1 [file medicina-62-00783-s001.zip › Supplementary Table S3. Quality.pdf]

Quality assessment results- Supplementary Table S3.

To evaluate the 21 studies using the Cochrane Risk of Bias tool, the Newcastle-Ottawa Scale, and ROBINS-I, a summary table was created that included the following:

1. Study Title: Reference to the study title.
2. Study Type: Cross-sectional, Prospective, Cohort, Retrospective, Randomized Clinical Trial, etc.
3. Cochrane Risk of Bias (RoB): Assessment for randomized studies (low, moderate, high risk).
4. Newcastle-Ottawa Scale (NOS): Score for cohort and observational studies (1–9).
5. ROBINS-I: Assessment for non-randomized studies (low, moderate, serious, critical risk).

Table S3. Assessment of studies based on the Cochrane RoB, NOS, and ROBINS-I assessment tools

Study Title	Study type	Cochrane RoB	NOS (1-9)	ROBINS-I
Bamber et al. (2013)	Randomized Clinical Trial	Low	-	-
Bartoli-Abdou et al. (2018)	Cross-sectional	-	7	Moderate
Brekelmans et al. (2017)	Cross-sectional	-	6	Moderate
Cajfinger et al. (2016)	Prospective	-	8	Moderate
Cano et al. (2018)	Registry-based observational study	-	7	Moderate
Dault et al. (2018)	Cross-sectional	-	6	Moderate

Study Title	Study type	Cochrane RoB	NOS (1-9)	ROBINS-I
Fang et al. (2022)	Cohort study	-	8	Moderate
Farge et al. (2019)	Prospective	-	8	Moderate
Font et al. (2023)	Prospective	-	7	Moderate
Haac et al. (2017)	Pilot study	-	6	Moderate
Hendriks et al. (2020)	Cohort study	-	7	Moderate
Hull et al. (2009)	Randomized Clinical Trial	Low	-	-
Keita et al. (2017)	Cross-sectional	-	6	Moderate
Lutsey et al. (2023)	Cross-sectional	-	6	Moderate
Maraveyas et al. (2020)	Cohort study	-	8	Moderate
Noble et al. (2022)	Cohort study	-	7	Moderate
Picker et al. (2021)	Cohort study	-	7	Moderate
Prins et al. (2018)	Randomized Clinical Trial	Low	-	-
Schulman et al. (2017)	Registry-based observational study	-	7	Moderate
Webb et al. (2019)	Cross-sectional	-	6	Moderate
Wong et al. (2010)	Cross-sectional	-	6	Moderate

Explanation and commentary on the study assessment

1.Cochrane RoB: Applies only to randomized clinical trials. Most studies had a low risk of bias due to good design.
2. Newcastle-Ottawa Scale (NOS): Applies to cohort and observational studies. Most studies scored between 6 and 8, indicating moderate to good quality.
3. ROBINS-I: Applies to non-randomized studies. Moderate risk was the most common due to potential confounding factors
