# Supplementary material for: Patient Satisfaction with Anticoagulation for Venous Thromboembolic Disease: A Systematic Review of Oral and Parenteral Regiments
Source: Medicina (Kaunas). 2026 Apr 17;62(4):783. doi: 10.3390/medicina62040783 (PMC13117933; doi:10.3390/medicina62040783)
Supplement: Supplementary file 1 [file medicina-62-00783-s001.zip › Supplementary Table S1. PRISMA 2020 Checklist.pdf]

Table S1: PRISMA 2020 Checklist for the study: "Patient Satisfaction with Anticoagulation for Venous Thromboembolic Disease: A systematic Review of Oral and Parenteral Regiments

Title and Abstract

Section and Topic	Item	Checklist item	Location where item is reported
Title	1	Identify the report as a systematic review.	Title: **"Patient Satisfaction with Anticoagulation for Venous Thromboembolic Disease: A systematic Review of Oral and Parenteral Regiments" (**Title page).
Abstract	2	See the PRISMA 2020 for Abstracts checklist.	Abstract section (Background/Introduction, Objectives/Aim, Materials and Methods, Results, Conclusions, Keywords).

Introduction

Section and Topic	Item	Checklist item	Location where item is reported
Rationale	3	Describe the rationale for the review in the context of existing knowledge.	Introduction – first paragraphs on burden, morbidity/mortality, economic impact, complications (PTS, CTEPH), and link to patient satisfaction and anticoagulation options.
Objectives	4	Provide an explicit statement of the objective(s) or question(s) the review addresses.	Introduction – last paragraph: primary aim to evaluate patient satisfaction with anticoagulation in VTE and impact on QoL; secondary objectives (factors influencing satisfaction, comparison of standard treatments vs DOACs, relation to patient-reported outcomes).

Methods

Section and Topic	Item	Checklist item	Location where item is reported
Eligibility criteria	5	Specify the inclusion and exclusion criteria for the review and how studies were grouped for the syntheses.	Methods – "Inclusion and Exclusion Criteria in the Study": adult population ≥18 years, English language, VTE with anticoagulation satisfaction, study designs (RCTs, observational, prospective cohorts), use of weighted instruments; detailed exclusion criteria (pregnant women, AF-only, mixed AF+VTE, other indications, reviews, case reports/series, no satisfaction measures, no weighted tools).
Information sources	6	Specify all databases, registers, websites, organisations, reference lists and other sources searched or consulted to identify studies. Specify the date when each source was last searched or consulted.	Methods – "Methodology/2. Methodology": PubMed, EMBASE, Cochrane Library, Google Scholar, CINAHL, Web of Science; additional sources: Neapolis University Library, HFAISTOS repository, Europeana; period 2009 to December 2024. PRISMA flow diagram text with numbers (Figure 1).
Search strategy	7	Present the full search strategies for all databases, registers and websites, including any filters and limits used.	Methods – "Methodology/2. Methodology": lists key terms ("venous thromboembolic disease", "deep vein thrombosis", "pulmonary embolism", "oral anticoagulation", "vitamin K antagonists", "direct oral anticoagulants", "patient satisfaction", "treatment satisfaction", "quality of life-QoL") and their combinations. No full line-by-line database strategy reported.
Selection process	8	Specify the methods used to decide whether a study met the inclusion criteria of the review, including how many reviewers screened each record and each report retrieved, whether they worked independently, and if applicable, details of automation tools used in the process.	Methods – "Selection of Studies – Prisma Diagram" and PRISMA flow diagram (Figure 1): numbers identified, screened, excluded, and included; reasons for exclusion (language, AF-only, mixed populations, pregnancy, different primary objective, HRQoL from disease not treatment, focus on care setting). Number of

Section and Topic	Item	Checklist item	Location where item is reported
			reviewers/independence not explicitly detailed.
Data collection process	9	Specify the methods used to collect data from reports, including how many reviewers collected data from each report, whether they worked independently, any processes for obtaining or confirming data from study investigators, and if applicable, details of automation tools used in the process.	Methods – “Export of Data and Assessment of the Quality of Studies”: describes construction of a standardized data-extraction form (study characteristics, demographics, regimens, tools, outcomes, follow-up). Number of reviewers/independence and contact with authors not explicitly reported.
Data items	10a	List and define all outcomes for which data were sought. Specify whether all results that were compatible with each outcome domain in each study were sought (e.g. for all measures, time points, analyses), and if not, the methods used to decide which results to collect.	Methods – “Export of Data and Assessment of the Quality of Studies” and “Synthesis and Analysis of Data”: outcomes include satisfaction scores, QoL measures, primary and secondary outcomes, success rates, adverse event rates, adherence, patient preferences; results standardized to compare tools.
Data items	10b	List and define all other variables for which data were sought (e.g. participant and intervention characteristics, funding sources). Describe any assumptions made about any missing or unclear information.	Methods – “Export of Data and Assessment of the Quality of Studies”: data on study design, number of patients, duration, location, setting, demographic data, anticoagulation regimens (type, dose, route), follow-up duration. Assumptions for missing/unclear data not explicitly discussed.
Study risk of bias assessment	11	Specify the methods used to assess risk of bias in the included studies, including details of the tool(s) used, how many reviewers assessed each study and whether they worked independently, and if applicable, details of	Methods – “Export of Data and Assessment of the Quality of Studies”: tools listed – Cochrane Risk of Bias for RCTs, Newcastle–Ottawa scale for observational studies, ROBINS-I for non-randomized interventional studies. Number of assessors and independence not specified.

Section and Topic	Item	Checklist item	Location where item is reported
		automation tools used in the process.	
Effect measures	12	Specify for each outcome the effect measure(s) (e.g. risk ratio, mean difference) used in the synthesis or presentation of results.	Methods – "Synthesis and Analysis of Data": satisfaction scores standardized; meta-analyses with random-effects models; success and adverse event rates summarized (percentages, ranges). Specific statistical effect measures per outcome (e.g. risk ratio, mean difference) not always explicitly named but implied in quantitative synthesis and meta-analyses.
Synthesis methods	13a	Describe the processes used to decide which studies were eligible for each synthesis (e.g. tabulating the study intervention characteristics and comparing against the planned groups for each synthesis (item #5)).	Methods – "Synthesis and Analysis of Data" and Results – "Patient Demographics", "Measurement Tools for Patient Satisfaction", "Results of the Systematic Review…", "Comparison between Treatments": studies grouped by type of anticoagulant (DOACs, VKAs, LMWHs), by measurement tools, and by clinical population (e.g. cancer-associated VTE).
Synthesis methods	13b	Describe any methods required to prepare the data for presentation or synthesis, such as handling of missing summary statistics, or data conversions.	Methods – "Synthesis and Analysis of Data": satisfaction scores standardized across different instruments to allow comparison; handling of missing summary statistics not detailed.
Synthesis methods	13c	Describe any methods used to tabulate or visually display results of individual studies and syntheses.	Methods – "Synthesis and Analysis of Data" and Results – Tables 1–4: tabulation of demographics (Table 1), measurement tools (Table 2), satisfaction by treatment type and influencing factors (Tables 3–4); narrative and tabular summaries.
Synthesis methods	13d	Describe any methods used to synthesize results and provide a rationale for the choice(s). If meta-analysis was performed, describe the model(s),	Methods – "Synthesis and Analysis of Data": qualitative and quantitative analyses; meta-analyses performed using random-effects models to account for heterogeneity; I^2 statistic

Section and Topic	Item	Checklist item	Location where item is reported
		method(s) to identify the presence and extent of statistical heterogeneity, and software package(s) used.	used to assess heterogeneity. Statistical software not specified.
Synthesis methods	13e	Describe any methods used to explore possible causes of heterogeneity among study results (e.g. subgroup analysis, meta-regression).	Methods – “Synthesis and Analysis of Data”: subgroup analyses planned/performed by type of anticoagulation (DOACs vs VKAs), duration of treatment, type of VTE (DVT vs PE), and geographical area. No meta-regression reported.
Synthesis methods	13f	Describe any sensitivity analyses conducted to assess robustness of the synthesized results.	Methods/Results: Sensitivity analyses are not explicitly described; item effectively not reported.
Reporting bias assessment	14	Describe any methods used to assess risk of bias due to missing results in a synthesis (arising from reporting biases).	Methods – “Synthesis and Analysis of Data”: publication bias assessed using funnel plots and Egger's test.
Certainty assessment	15	Describe any methods used to assess certainty (or confidence) in the body of evidence for an outcome.	No formal GRADE or other certainty-of-evidence framework described; item not reported.

Results

Section and Topic	Item	Checklist item	Location where item is reported
Study selection	16a	Describe the results of the search and selection process, from the number of records identified in the search to the number of studies included in the review, ideally using a flow diagram.	Methods – “Selection of Studies – Prisma Diagram” and Figure 1; PRISMA 2020 flow diagram document: 539 records from databases plus registers; duplicates and ineligible records removed; 294 screened; 164 reports sought; 106 assessed; 21 studies included (50 reports).
Study selection	16b	Cite studies that might appear to meet the inclusion criteria, but	Methods – “Selection of Studies – Prisma Diagram”

Section and Topic	Item	Checklist item	Location where item is reported
		which were excluded, and explain why they were excluded.	and PRISMA flow text: categories of excluded reports (non-English/Greek, reviews, AF-only, mixed AF+VTE, pregnancy, different primary objective, HRQoL from disease not treatment, focus on care setting). Individual excluded studies not cited by name.
Study characteristics	17	Cite each included study and present its characteristics.	Results – “3. Patient Demographics” (Table 1) lists individual studies (e.g. Wong et al. 2014; Bamber et al. 2013; Bartoli-Abdou et al. 2018; Brekelmans et al. 2017; Cajfinger et al. 2016; Cano et al. 2018; Dault et al. 2018; Fang et al. 2022) with number of patients, sex, mean age, condition, treatment; additional study details and full citations in References.
Risk of bias in studies	18	Present assessments of risk of bias for each included study.	Methods describe tools, but the Results section does not present individual or summarized risk-of-bias assessments (no dedicated table/figure); item not reported in results.
Results of individual studies	19	For all outcomes, present, for each study: (a) summary statistics for each group (where appropriate) and (b) an effect estimate and its precision (e.g. confidence/credible interval), ideally using structured tables or plots.	Results – narrative synthesis and Tables 1–4: descriptive data, satisfaction proportions (e.g. ~80% satisfied overall, success and adverse event rates, qualitative satisfaction by therapy type). Individual study-level effect estimates with CIs are not systematically tabulated.
Results of syntheses	20a	For each synthesis, briefly summarise the characteristics and risk of bias among contributing studies.	Results – “5. Results of the Systematic Review of Patient Satisfaction with Anticoagulation Therapy for

Section and Topic	Item	Checklist item	Location where item is reported
			VTE” and “6. Comparison between Treatments”, “7. Key Factors and Patient Preferences”: summarize characteristics of included studies, patient numbers, age, sex distribution, therapy types, tools used; risk of bias not summarized here.
Results of syntheses	20b	Present results of all statistical syntheses conducted. If meta-analysis was done, present for each the summary estimate and its precision (e.g. confidence/credible interval) and measures of statistical heterogeneity. If comparing groups, describe the direction of the effect.	Results – quantitative summary: overall ~80% satisfaction; success rate 88.9% (range 60.4–96%); adverse events 5.6% (range 2–12%); higher satisfaction with DOACs vs VKAs and LMWHs; DOACs associated with higher QoL and adherence; some reference to meta-analyses in text. Pooled effect sizes with confidence intervals and I^2 values are not explicitly tabulated.
Results of syntheses	20c	Present results of all investigations of possible causes of heterogeneity among study results.	Results – “5. Results…”, “6. Comparison between Treatments”, “7. Key Factors and Patient Preferences”, “8. Discussion”: exploration of heterogeneity by treatment type (DOACs vs VKAs vs LMWHs), cancer vs non-cancer, route of administration, monitoring needs, age group, sex, and geographical setting; described narratively.
Results of syntheses	20d	Present results of all sensitivity analyses conducted to assess the robustness of the synthesized results.	Sensitivity analyses are not explicitly presented; item not reported.
Reporting biases	21	Present assessments of risk of bias due to missing results (arising from reporting biases) for each synthesis assessed.	Publication bias is mentioned as assessed with funnel plots and Egger's test in Methods; specific results of these

Section and Topic	Item	Checklist item	Location where item is reported
			assessments are not separately reported in the Results section; item partially reported.
Certainty of evidence	22	Present assessments of certainty (or confidence) in the body of evidence for each outcome assessed.	No formal certainty-of-evidence (e.g. GRADE) assessment reported; item not reported.

Discussion and Other Information

Section and Topic	Item	Checklist item	Location where item is reported
Discussion	23a	Provide a general interpretation of the results in the context of other evidence.	Discussion (section "8. Discussion" and subsequent paragraphs): interprets ~80% high satisfaction, superiority of DOACs in convenience and QoL, role of route of administration, monitoring burden, and alignment with external evidence (e.g. COSIMO, SWAN, XALIA, cancer-associated VTE and guideline papers).
Discussion	23b	Discuss any limitations of the evidence included in the review.	Discussion (throughout, especially paragraphs discussing heterogeneity, limited number of VTE-specific satisfaction studies, varying tools, and differences in populations and settings). Limitations of available evidence are described qualitatively.
Discussion	23c	Discuss any limitations of the review processes used.	Discussion: implicit comments on heterogeneity of tools and designs; explicit limitations of the review methods (e.g. language restriction, possible publication bias, lack of registration) are not fully elaborated; item only partially addressed.
Discussion	23d	Discuss implications of the results for practice, policy, and future research.	Discussion and closing paragraphs: emphasize need to integrate patient satisfaction and preferences in

Section and Topic	Item	Checklist item	Location where item is reported
			guidelines and clinical decision-making, importance of patient education and shared decision-making, need for development of better satisfaction/QoL tools and more studies, and the shift toward patient-centred care.
Registration and protocol	24a	Provide registration information for the review, including register name and registration number, or state that the review was not registered.	Other Information – end of manuscript: no registration (e.g. PROSPERO) is reported; effectively "review was not registered".
Registration and protocol	24b	Indicate where the review protocol can be accessed, or state that a protocol was not prepared.	Other Information – no protocol reference given; implicitly indicates that a formal protocol was not prepared or not publicly available.
Registration and protocol	24c	Describe and explain any amendments to information provided at registration or in the protocol.	Not applicable – no registered protocol reported.
Support	25	Describe sources of financial or non-financial support for the review, and the role of the funders or sponsors in the review.	"Funding" statement in Other Information: support from the University of Thessaly Grants for Scientific Publication Support and Special Account of Research Grants; APC covered; funders' role described as funding framework, not influencing scientific content.
Competing interests	26	Declare any competing interests of review authors.	"Conflicts of Interest" statement: authors declare no conflict of interest.
Availability of data, code and other materials	27	Report which of the following are publicly available and where they can be found: template data collection forms; data extracted from included studies; data used for all analyses; analytic code; any other materials used in the review.	"Data Availability Statement": data presented in the study are available within the article; no separate public repository for data, forms, or code reported.
