# Supplementary material for: Patient Satisfaction with Anticoagulation for Venous Thromboembolic Disease: A Systematic Review of Oral and Parenteral Regiments
Source: Medicina (Kaunas). 2026 Apr 17;62(4):783. doi: 10.3390/medicina62040783 (PMC13117933; doi:10.3390/medicina62040783)
Supplement: Supplementary file 1 [file medicina-62-00783-s001.zip › Supplementary Table S2. PUBMED.pdf]

Supplementary Table S2. PubMed search strategy

Database: PubMed

Date of last search: December 2025

Limits: Humans, Adults (≥18 years), English language, Publication date from 1 January 2009 to 31 December 2025

Search string (PubMed):

(“venous thromboembolism”[Mesh] OR “venous thromboembolism”[tiab] OR “venous thromboembolic disease”[tiab] OR “VTE”[tiab] OR “deep vein thrombosis”[Mesh] OR “deep vein thrombosis”[tiab] OR “DVT”[tiab] OR “pulmonary embolism”[Mesh] OR “pulmonary embolism”[tiab] OR “PE”[tiab]) AND (“Anticoagulants”[Mesh] OR anticoagulant*[tiab] OR “oral anticoagulation”[tiab] OR “oral anticoagulant*”[tiab] OR “vitamin K antagonist*”[tiab] OR “VKA”[tiab] OR warfarin[tiab] OR acenocoumarol[tiab] OR “direct oral anticoagulant*”[tiab] OR DOAC*[tiab] OR rivaroxaban[tiab] OR apixaban[tiab] OR edoxaban[tiab] OR dabigatran[tiab] OR “low molecular weight heparin*”[tiab] OR LMWH[tiab]) AND (“Patient Satisfaction”[Mesh] OR “patient satisfaction”[tiab] OR “treatment satisfaction”[tiab] OR “Perception of Anticoagulant Treatment Questionnaire”[tiab] OR PACT-Q[tiab] OR “Anti-Clot Treatment Scale”[tiab] OR ACTS[tiab] OR “quality of life”[Mesh] OR “quality of life”[tiab] OR QoL[tiab]) AND (humans[Mesh])

Filters applied:

Publication date from 2009/01/01 to 2025/12/31

English language

Adults (≥18 years)
